# Synergetic effect of phosphonium-based ionic liquid and amine-functionalized metal organic framework for separation of CO_2_ and CH_4_ using mixed matrix membranes

**DOI:** 10.1038/s41598-026-48922-4

**Published:** 2026-04-13

**Authors:** Anil Mundhe, Sarthak Patel, Remya Ranjith, Tushar Patil, Ankush Bindwal, Rama Rao Karri, Swapnil Dharaskar

**Affiliations:** 1 CO2 Research Group, Department of Chemical Engineering, School of Energy Technology, Pandit Deendayal Energy University, Gandhinagar, 382426 India; 2 R&D Manager, Apar Industries Limited, Rabale, Navi Mumbai, Maharashtra 400701 India; 3 Distillate & Heavy Oil Processing Division, CSIR - Indian Institute of Petroleum, Dehradun, 248005 India; 4 Chemical and Energy Engineering, Faculty of Engineering, Universiti Teknologi Brunei, BE1410 Bandar Seri Begawan, Brunei Darussalam

**Keywords:** CO_2_, Metal organic framework, Ionic liquid, Mixed matrix membrane, Gas separation, Chemistry, Energy science and technology, Engineering, Environmental sciences, Materials science

## Abstract

Fossil fuels have emerged as one of the main sources of energy for human civilization in recent times. Greenhouse gas emissions are the result of numerous industries’ excessive dependence on fossil fuels as a source of energy. According to several studies and statistics, the atmosphere’s CO_2_ concentration is close to 422.80 parts per million. Additionally, the World Meteorological Organization reports that the CO_2_ concentration is increasing by 2 ppm annually. This work explains integrated functionalized metal-organic frameworks and ionic liquids into polyether-block-amide (Pebax-1657) mixed matrix membranes to improve CO_2_ permeability and CO_2_/CH_4_ selectivity. Various filler loadings of 5%, 10%, and 15% (relative to the polymer) were produced. Fourier transform infrared spectroscopy (FTIR), thermogravimetric analysis, X-ray diffraction (XRD), and scanning electron microscopy (SEM) were employed to ascertain the surface and cross-sectional morphology, characteristics, and functional groups of the metal-organic framework and the fabricated membranes. Permeation investigations utilizing both single-gas and mixed-gas methods were conducted for each membrane. The engineered membranes demonstrated remarkable efficacy, attaining a CO_2_ permeability of 226.37 Barrer and a selectivity of 25.4 for the CO_2_/CH_4_ mixture, thereby presenting new opportunities for improved CO_2_ separation and effective carbon capture.

**Supplementary Information:**

The online version contains supplementary material available at 10.1038/s41598-026-48922-4.

## Introduction

 The most urgent worldwide issue is climate change, which is mostly caused by emissions of greenhouse gases, especially carbon dioxide (CO_2_). Rising sea levels, melting glaciers, acidity of the ocean, and temperature increases are some of the effects. The primary cause of climate change and global warming is CO_2_. According to several studies and statistics by the National Oceanic and Atmospheric Administration (NOAA), as of January 2024, the atmosphere’s CO_2_ concentration is close to 422.80 parts per million. To avoid such calamities, various materials and techniques must be developed to reduce the concentration of CO_2_. Gas separation via membrane technology is a good candidate for this purpose among other carbon capture technologies, namely, adsorption, absorption, and cryogenic distillation, etc., due to several basic engineering and financial benefits of membranes, including low energy consumption, mild operating conditions, mobility, compactness, continuous separation, simplicity, and configurable characteristics^1,2^. The polymer membranes are inexpensive, easily scalable, simple to fabricate, and stable at high pressure. Rubber and glassy polymers are two categories into which the polymer membranes can be divided. Any gas separation membrane should have a balance between its gas permeation and selectivity, which means that when one parameter rises, the other one falls. The separation performance of inorganic membranes is generally better when compared to polymeric membranes, but they face difficulty in fabrication and processing due to their high brittleness and processibility^3^. The introduction of a novel membrane type called mixed matrix membranes (MMM), which have been researched ever since, was discovered in the 1970s^4^, using zeolite 5 A in Polydimethylsiloxane, resulting in a longer diffusion delay for CO_2_ and methane (CH_4_), which was one strategy for addressing the constraints that result from the trade-off nature of polymeric membranes. This hybrid membrane’s continuous phase is often an organic polymer matrix, while its dispersed phase is typically an inorganic filler.

In a nutshell, MMM simultaneously overcomes the drawbacks of inorganic materials and polymers as membranes while using their good qualities. Using a block polymer, which consists of both the glassy and rubbery polymer segments instead of either rubbery or glassy polymer, leads to better gas separation because the synergetic effect of both polymer types is that the rubbery polymer leads to high gas permeability and the glassy polymer leads to high selectivity. Commercially available Pebax^®^, also known as polyether-block-amide, is a block polymer of polyamide (PA) and polyether oxide (PEO). Particularly, Pebax-1657 consists of 60% glassy PA and 40% rubbery PEO, which results in better gas separation. Various fillers, such as Zeolites, covalent organic frameworks, carbon molecular sieves, carbon nanotubes, activated carbon, silica, metal oxides, and fullerene, are examples of both porous and nonporous fillers that can be used in MMM^5,6^.

Metal–Organic Frameworks (MOFs) are porous crystalline materials formed by metal centers linked with organic ligands. Their features—such as high surface area, tunable pore size, flexible functionalization, and structural stability—make them ideal fillers for MMMs. Among the UiO series (UiO-66, UiO-67, UiO-68), UiO-66, composed of benzene-1,4-dicarboxylate (BDC) and Zr_6_O_4_(OH)_4_ clusters, exhibits outstanding chemical stability and a surface area of 1000–1180 m^2^/g. Amine-functionalized form, UiO-66-NH_2_, though possessing a lower surface area, shows a stronger affinity toward polar gases like CO_2_, improving CO_2_/CH_4_ separation efficiency. There are several examples available in the literature of MOF-based MMMs for gas separation. Veetil et al.^7^ used 6FDA-durene and two different fillers, UiO-66-NH_2_ and PEG modified UiO-66-NH_2_, to MMMs. The first filler, UiO-66-NH_2_, had the largest permeability to CO_2_ of 2558.92 Barrer and the highest selectivity, 16.63. But when PEG was added to the filler known as PEG-modified UiO-66-NH_2,_ a distinct result was seen. The MMMs in this instance had an ideal selectivity of 22.40, which was higher than the selectivity attained with UiO-66-NH_2_. It’s important to remember that to obtain this better selectivity, CO_2_ permeability was reduced by about 53.13% as compared to UiO-66-NH_2_. The synergistic impact of 1-D and 3-D nanofillers on a MMM’s separation performance was examined by Khan et al.^8^. In order to decorate 3-D MOF (UiO-66-NH_2_) for this investigation, the researchers employed a polymer matrix of PSF and added 1-D amine-functionalized MWCNT as filler material into the polymer matrix. They investigated the MMM for the gas pair separation of CO_2_ and CH_4_. It is detected that with an increment in filler wt%, the value of gas permeability also increases. The MMM with 5.0 wt% UiO-66@MWCNT showed better permeability (8.3 Barrer) for pure CO_2_ than a pristine PSF membrane (4.2 Barrer).

Furthermore, the same MMM’s selectivity (CO_2_/CH_4_) increased to 39.5 from 28.0 for pure PSF under identical operational conditions. Zhang et al.^9^ investigated the gas separation of CO_2_ and CH_4_ by optimising several MOF blends using a PI-based MMM. They incorporated MOF into the mixture after altering the polymer matrix using an ionic liquid (IL). With a good selectivity of 74.1 for CO_2_/CH_4_, the modified PI membrane by IL demonstrated 6.2 times the selectivity of the pure PI membrane. By using MOF, the permeability and selectivity were both improved. For instance, a MMM with a 3 wt% loading of UiO-66-NH_2_ demonstrated a roughly 700% rise in CO_2_/CH_4_ selectivity and a roughly 539% increase in selectivity, both of which are near the 2008 Robeson upper bound. They concluded that the notable improvement was caused by the addition of MOF and IL. To improve the CO_2_ separation and anti-aging properties of the membrane, Gao et al.^10^ developed a variety of MMMs. In their work, they employed amidoxime-modified UiO-66 frameworks (UiO-66-AO) as the filler and PIM-1 as the polymer matrix. The gas separation study includes gases like CO_2_, CH_4,_ and N_2_. The 20 wt% loaded MMM yielded the best overall performance. Comparing the CO_2_/CH_4_ selectivity to the pristine PIM-1 membrane, there was an approximate 47% enhancement and a 50% increase in CO_2_ permeability. The findings demonstrate that the inclusion of UiO-66-AO significantly enhanced the membrane’s anti-aging characteristics. Despite extensive studies on MOF-based MMMs, limited work has explored the synergistic integration of UiO-66-NH_2_ with ionic liquids like [THTDP][NTf_2_] in Pebax-1657 matrices. The combined influence of MOF loading and IL interaction on membrane morphology, gas transport, and long-term stability for CO_2_/CH_4_ separation remains insufficiently understood. [THTDP][NTf2] was selected due to its high thermal stability, hydrophobic nature, low volatility, and strong CO_2_ affinity associated with the NTf_2_⁻ anion. Compared to many imidazolium-based ILs, phosphonium ILs exhibit enhanced chemical stability and compatibility with polymer matrices, making them suitable for mixed matrix membrane fabrication.

In this study, MMMs were fabricated using Pebax-1657, UiO-66-NH_2,_ and Trihexyl tetradecyl phosphonium bis (trifluoromethyl sulfonyl) imide ([THTDP][NTf_2_]). Different MMMs were created using varying filler loadings of UiO-66-NH_2_ from 5 to 15 weight% based on polymer. The surface and cross-sectional morphology, properties, and functional groups of the manufactured membranes are determined by characterizations such as Fourier transform infrared spectroscopy (FTIR), x-ray diffraction (XRD), thermogravimetric analysis (TGA), and scanning electron microscopy (SEM). Single as well as mixed gas studies have been performed to understand the gas transport phenomena through the membrane. Additionally, the impact of UiO-66-NH_2_ loading and trans membrane pressure effect on the membrane properties is investigated.

## Experimental

### Materials

The study utilised chemicals from Arkema specialty polyamides, specifically Pebax-1657, for the manufacture of the membrane. [THTDP][NTf_2_] is the IL that was purchased from Sigma Aldrich, India, along with organic solvents and precursors like ethanol, dimethyl formaldehyde (DMF), acetic acid, 2-amino terephthalic acid (NH_2_-BDC), and zirconyl chloride octahydrate (ZrOCl_2_.8H_2_O) (> 98%purity). Every chemical and solvent used was of analytical grade. We acquired a porous polyvinylidene fluoride (PVDF) membrane from Millipore, Merck. For the gas permeation study, Nishi Enterprise (Ahmedabad, India) provided CO_2_ (99.99%) and CH_4_ (99.99%). After acquiring it, the chemicals weren’t processed any further. A list of the compounds used in the current study, together with their properties, is provided in Table 1.

**Table 1:** Properties of chemicals used in this work.

Name	Abbreviation	CAS No.	MW (g/mol)	Melting point (ºC)	State	Purity (%)
Polyether block amide	Pebax-1657	–	~1438	204	Solid	≥ 98
Tri hexyl tetradecyl phosphonium bis (trifluoromethyl sulfonyl) imide	[THTDP][NTf_2_]	460092-03-9	763.24	−72	Liquid	≥ 95
2-aminoterephthalate; oxygen (2^−^); zirconium(4^+^);tetra hydroxide	UiO-66-NH_2_	10312-55-7	1754.1	–	Solid	–

### UiO-66-NH_2_ nanoparticle synthesis

In a standard synthesis, NH_2_BDC acid and ZrOCl_2_.8H_2_O were added in a 1:1 molar ratio. To create a homogeneous solution, NH_2_BDC (~ 3 mmol) and ZrOCl_2_.8H_2_O (~ 3 mmol) were both added to a 30 mL beaker containing DMF. The mixture was then agitated for 2 h at room temperature. The uniform mixture was then transferred to a Teflon-lined stainless-steel reactor. To promote crystal formation, a certain amount (2 ml) of acetic acid was added. The autoclave, along with the reaction mixture, was then kept in an oven at 150 °C for a whole day. UiO-66-NH_2_ particles were separated using a centrifuge at 10,000 rpm. The powder was then washed with DMF and methanol to remove unreacted precursors and impurities. Subsequently, the resultant material was dried for 18–20 h at 110 °C in a vacuum oven^11^.

### Fabrication of the membrane

MMMs are prepared by the solution casting method. The MMMs of Pebax-1657 consisting of UiO-66-NH_2_ (Pebax-1657 / UiO-66-NH_2_ / [THTDP][NTf_2_] MMMs) prepared with different loadings of UiO-66-NH_2_ (5, 10, and 15 wt%). A 15 wt% Pebax-1657 solution was prepared using a solvent composed of 70:30%v/v ethanol and deionized water. Pebax-1657 was entirely dissolved after 4 h of continuous stirring at 90 °C under reflux. Before casting, the mixture was thoroughly sonicated for 15–30 min to eliminate any bubbles that may have formed while stirring. After a uniform polymeric solution was created, it was cast using a digital casting blade onto a PVDF porous membrane. At room temperature, the solvent was evaporated for approximately 1 h. After that, the membrane was kept in an 80 °C vacuum oven for the entire night to eliminate the solvent^12^. The same procedure was used to create MMMs, but at first, 15 wt% (depending on the polymer loading [THTDP][NTf_2_] was dissolved rather than dissolving Pebax-1657. After the proper IL dissolution, different loadings of UiO-66-NH_2_ (depending on the concentration of the polymer) were added to accomplish total dispersion. The Pebax-1657 was dissolved after that. A homogenous polymeric solution was achieved by stirring it at 95 °C for 24 h. Subsequently, the dope solution was sonicated for 30 min to ensure complete removal of trapped bubbles, and then it was cast using a digital casting blade onto a PVDF porous membrane. At normal temperature, the solvent evaporated in roughly one hour. After that, the membrane was kept in an 80 °C vacuum oven for the entire night to eliminate the solvent^13^.

### UiO-66-NH_2_ and pebax / UiO-66-NH_2_ / [THTDP][NTF2] MMMs characterization

The existence of functional groups in UiO-66-NH_2_ and the membrane for wave numbers that span between the 4000 cm^− 1^ and 500 cm^− 1^ at room temperature was determined using FTIR-ATR spectroscopy (The ALPHA Spectrometer) with a resolution of 4 cm^− 1^. The crystalline behavior of the UiO-66-NH_2_ nanoparticles and MMMs was determined by XRD using a Rigaku Miniflex benchtop X-ray Diffractometer. HITACHI STA7400 was used to perform TGA of UiO-66-NH_2_ nanoparticles and membranes. The sample was heated in a N_2_ atmosphere at 35–800 °C for UiO-66-NH_2_, and for MMMs 35–600 °C at the rate of 10 °C per minute to obtain its temperature profile. Zeiss LSM 510 Meta was used to generate SEM and determine the morphology of the produced UiO-66-NH_2_. Additionally, the membrane surface, cross-sectional morphology, and distribution of UiO-66-NH_2_ in the polymer matrix were examined using JEOL JSM-7600 F. A required layer of gold was sputtered before putting them into SEM imaging.

### Gas permeation study

To determine whether Pebax-1657 / UiO-66-NH_2_ / [THTDP][NTf2] MMMs are suitable for gas separations, a gas permeation experiment was performed at different pressures varying from 1 to 4 bars. The membrane was cut into a circular shape of diameter 2.5 cm and used for a gas permeation study. Gas permeability and CO_2_/CH_4_ selectivity were determined using Eqs. 1 and 2:

1 $$\:P=\:\frac{{Q}_{i}L}{{\varDelta\:P}_{i}A}\:$$

2 $$\:{\alpha\:}_{\frac{C{O}_{2}}{C{H}_{4}}}=\:\frac{{P}_{{CO}_{2}}}{{P}_{{CH}_{4}}}\:$$

**Fig. 1:**
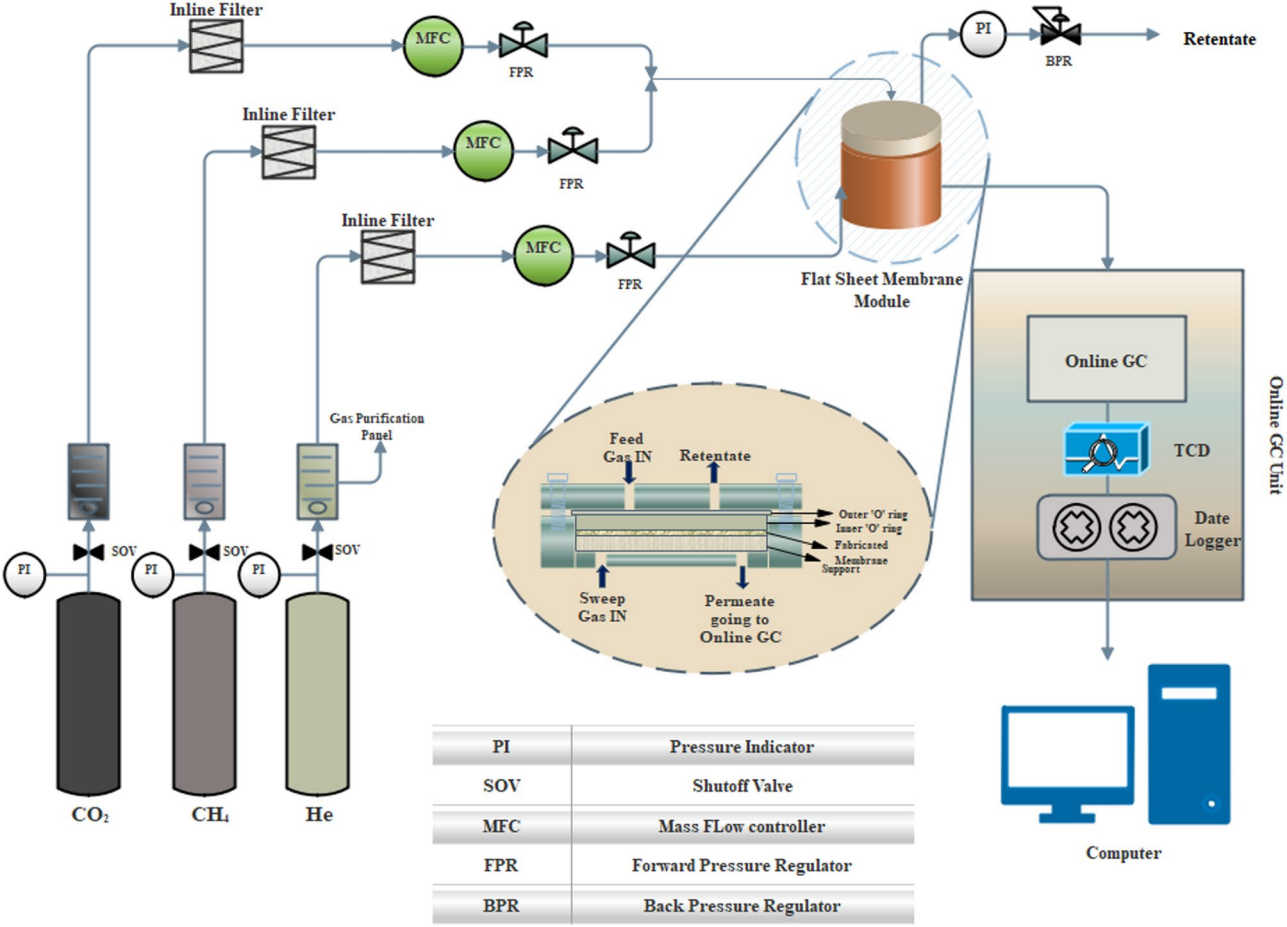
Gas permeation setup with online gas chromatograph.

Here, P represents the permeability of gas in Barrer, Q is the flow rate of gas per area (cm^3^/s), L denotes membrane thickness (cm), and A represents the area of membrane under study (cm^2^), $\:\varDelta\:P$ is the transmembrane pressure (cm Hg). It is important to note that in membrane studies, Barrer and bar are the standard units used to represent pressure and gas permeability. $\:{P}_{{CO}_{2}}$ is CO_2_ permeability, $\:{P}_{{CH}_{4}}$ is CH_4_ permeability, and $\:\alpha\:$ is the CO_2_/CH_4_ selectivity. To ensure the results could be repeated, each experiment was run three times. Figure 1 depicts the gas permeation setup that was used to perform the permeation experiments.

## Results and discussion

### Characterization of Uio-66-NH2 nanoparticle and membranes

#### FTIR

**Fig. 2:**
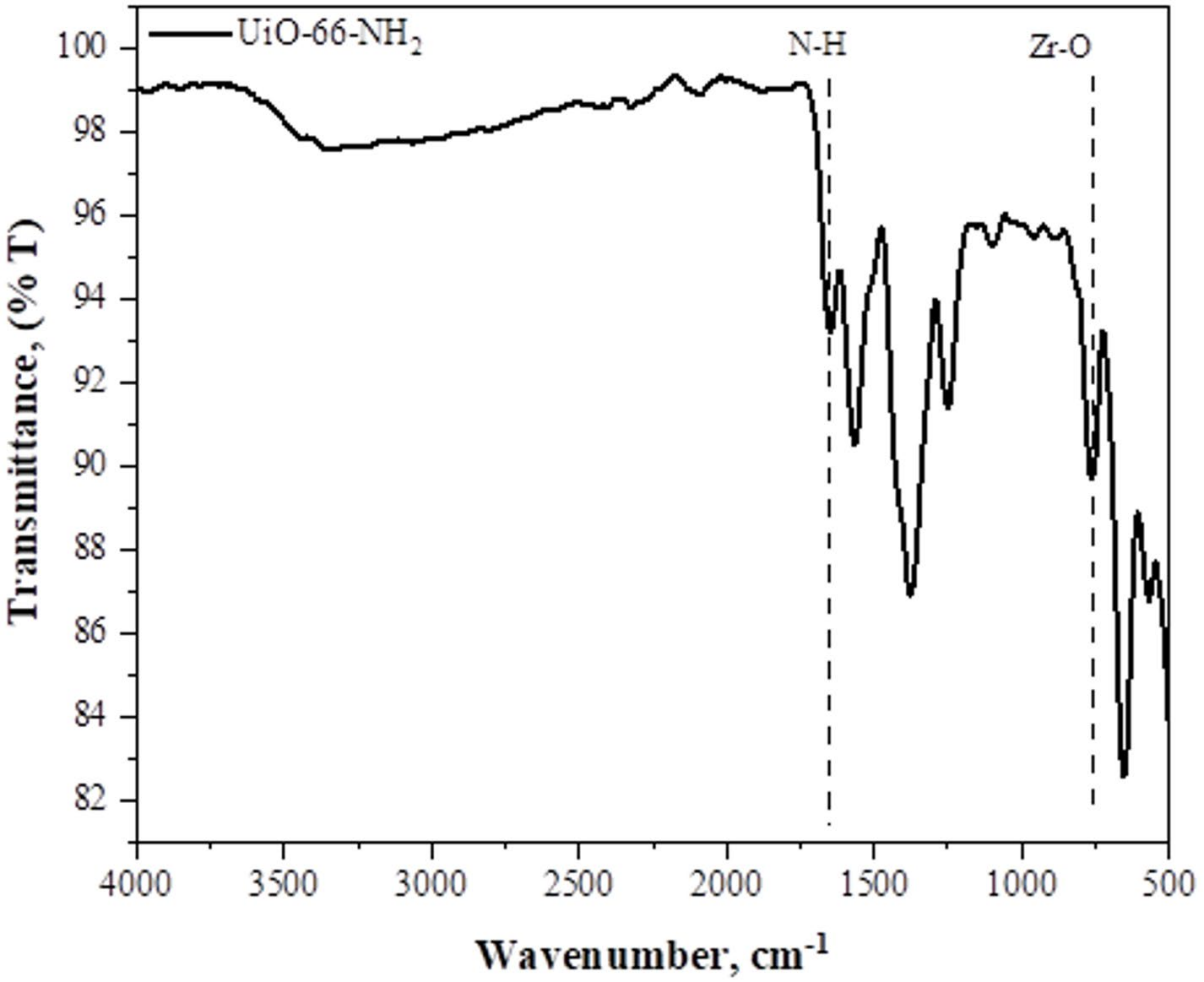
FTIR Spectra of UiO-66-NH_2_.

**Fig. 3:**
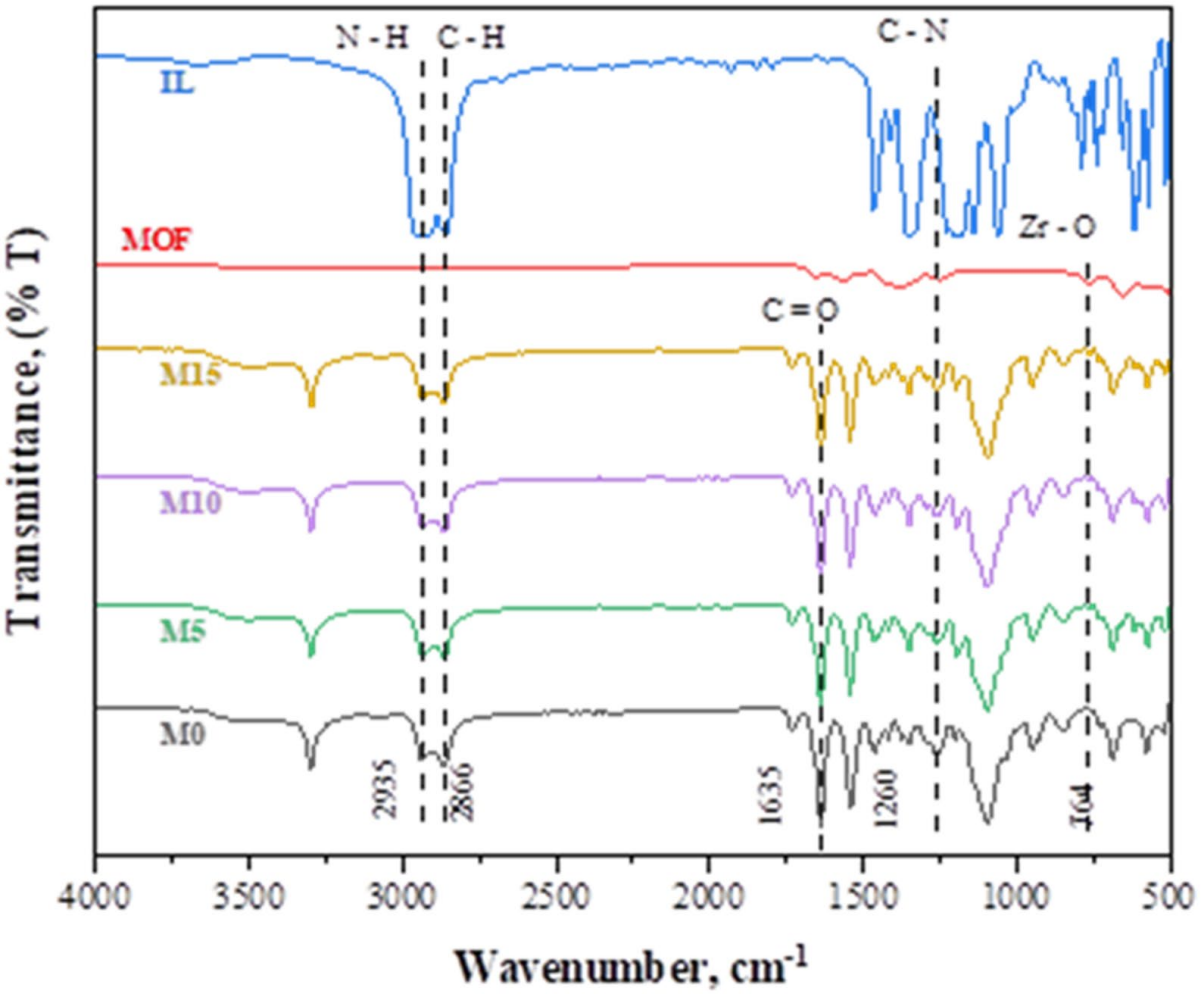
FTIR spectra of neat Pebax-1657, MMMs, [THTDP][NTf_2_].

Various kinds of chemical bonds that are present in a substance can be identified using FTIR. FTIR of the prepared UiO-66-NH2 and membranes is plotted in Figs. 2 and 3. In Fig. 2, the observed peak around 1653 cm^− 1^ is ascribed to the stretching of the amine (NH) bond, while sagging-like behavior is observed between 3000 cm^− 1^ and 3500 cm^− 1^, which is related to C–OH bond stretching^14^. With peaks at 1568 cm^− 1^ and 1380 cm^− 1^, respectively, this spectrum shows carbon to carbon or carbon to oxygen bond stretching. The presence of aromatic amine is confirmed by the peak at 1254 cm^− 1^. Zr-O is responsible for the peak found at 764 cm^− 1^^11^.

The characteristic peaks resemble the pristine membranes, as illustrated in Fig. 3. The PEO part present in Pebax shows a stretch due to vibration at 1095 cm^−1^. The peaks at 1543 cm^−1^ and 1635 cm^−1^ indicate the amine group and C = O groups in PA and PEO phases, respectively. The peak at 1733 cm^−1^ is due to the carbonyl group stretching in the polyamide section of the polymer. At wavenumber 764 cm^−1^, a peak is caused by the existence of the Zr-O bond, which can be used to validate the presence of UiO-66-NH_2_ in all MMMs^11^. The filler loading rises from 5 to 15 wt%, and this is accompanied by a slight rise in peak intensity. Because C-N groups of UiO-66-NH_2_ particles are present in MMMs, the amplitude of the stretching vibration peak corresponding to C-N at 1260 cm^−1^ does not vary as filler loading increases. The observation of a strong peak of 2935 cm^−1^ is ascribed to the amino group’s N-H bond vibrations.

#### XRD

**Fig. 4:**
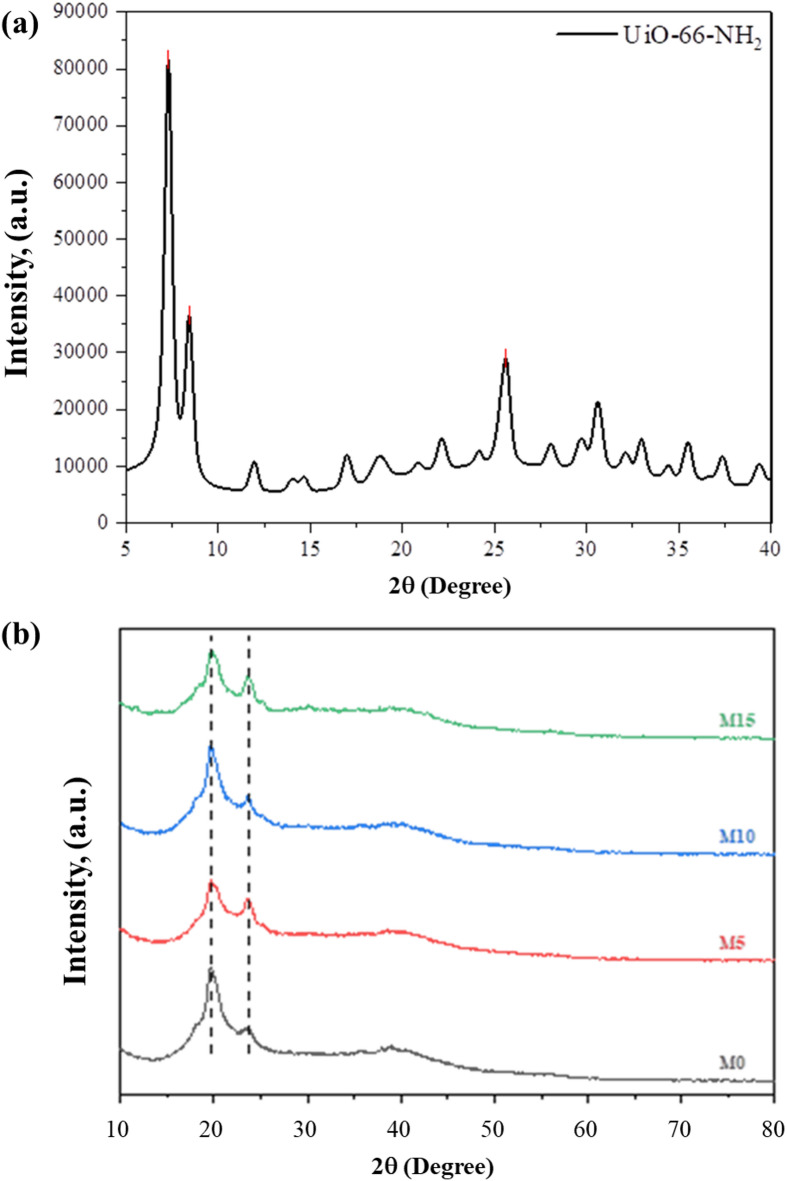
(**a**) XRD of UiO-66-NH2 MOF; (**b**) XRD of Membranes along with Neat Pebax.

The synthesized UiO-66-NH_2_s’ XRD patterns are shown in Fig. 4. In Fig. 4a.

It has been observed that a more crystalline structure is achieved during hydrothermal synthesis by using acid modulators, including hydrochloric acid, acetic acid, and formic acid^15,16^. The X-ray diffraction trend reveals the structure of UiO-66-NH_2_. The positions of all the diffraction peaks are the same as those previously synthesized^14,17^. The peak shift after the addition of the amine group has been indicated by a peak shift from 7.4° & 8.5° degrees to 7.31° and 8.43° degrees. The positions of the metal organic framework and the morphological alterations in the polymer were examined using the XRD patterns of MMMs and Pebax-1657, which are shown in Fig. 4b. Pebax-1657 is a semicrystalline copolymer having crystalline polyamide and amorphous polyether segments. Peaks in the range of 2θ = 16°2–5° degrees show both the segments. At about 2θ = 17°, additional peaks show up in the MMMs. Fillers may be the cause of this alteration. The intensity of the peak at 23° increases as the filler loading increases. Additionally, the polymer peak pattern slightly widened, suggesting that the UiO-66-NH_2_ disrupts the polymer’s chain packing, which leads to the creation of free volume for gas transport^18^. The low MOF loading and the dominant amorphous nature of the polymer matrix, which masked the crystalline reflections of the filler. The absence of the characteristic diffraction peaks of UiO-66-NH_2_ in the XRD pattern of the mixed matrix membrane is mainly due to the dominant amorphous nature of the polymer matrix and the relatively low loading of the MOF. The ionic liquid further increases the amorphous background, reducing the overall intensity of MOF reflections. In addition, uniform dispersion of small MOF particles within the polymer can decrease peak intensity and cause peak broadening. Therefore, the disappearance of UiO-66-NH_2_ peaks in the membrane XRD pattern does not necessarily indicate structural damage of the MOF, but rather results from dilution, peak overlap, and the strong contribution of the polymer matrix. Another plot of UiO-66-NH_2_ particle, along with membranes, has been plotted in Fig. S4.

#### TGA

**Fig. 5:**
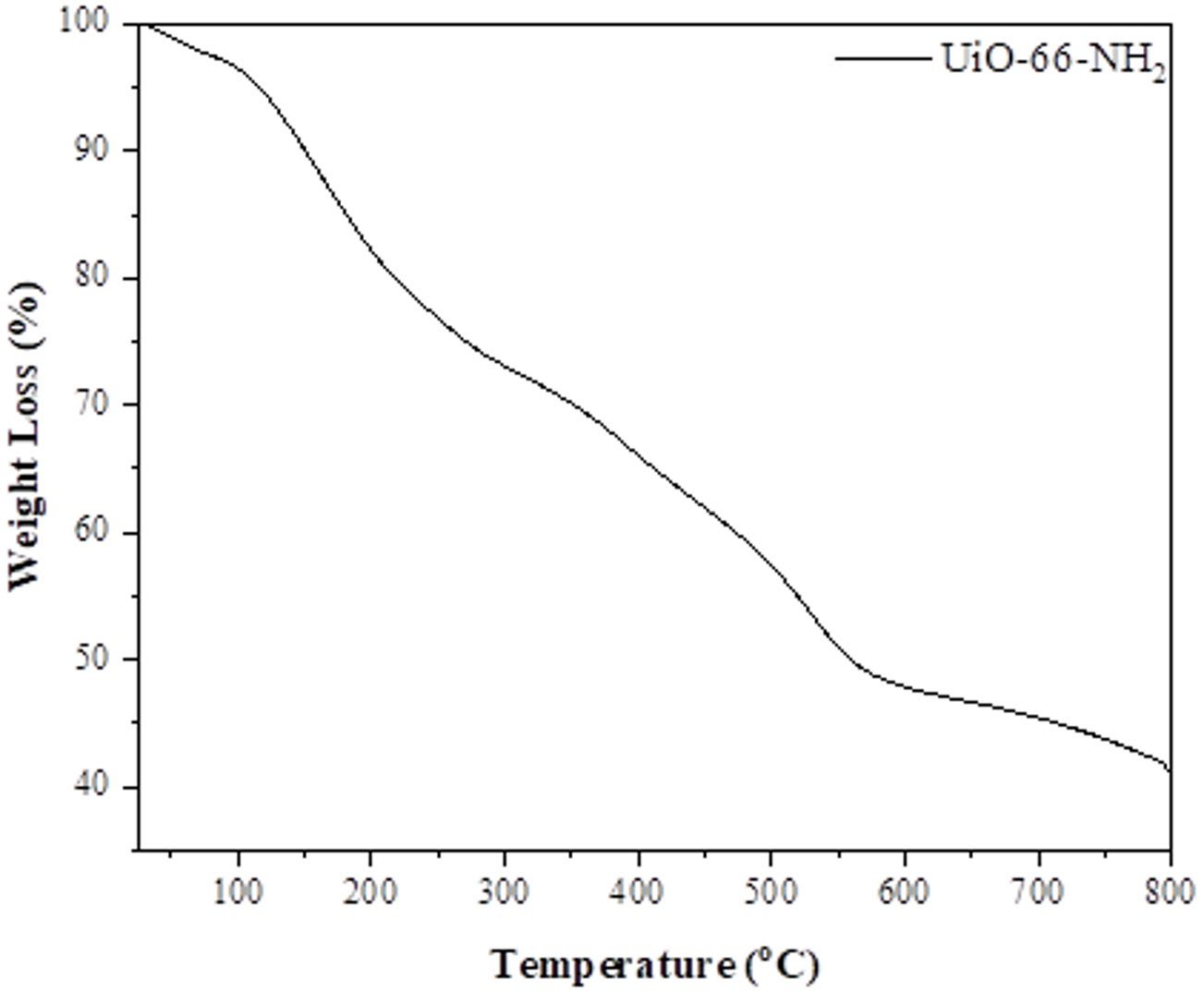
Thermo gravimetric analysis of UiO-66-NH_2_ *(N*_2_ *Atmosphere*,* Temperature 35-800*^*o*^*C*,* Scan rate-10*^*o*^ */min)*.

The temperature changes against the weight loss of UiO-66-NH_2_ MOF are depicted in Fig. 5 Moisture is the primary cause of the weight loss for UiO-66-NH_2_ between 35 °C and 110 °C. Between 270 °C and 400 °C, a larger slope is seen, which is due to the loss of excess solvent. The TGA graph for UiO-66-NH_2_ differs from UiO-66, based on the literature, having an additional stage in the 315–400 °C temperature range, which indicates the degradation of the amine groups. Final weight loss in UiO-66-NH_2_, which is explained by the dicarboxybenzene ligand breaking down at 400 °C^19,20^.

**Fig. 6:**
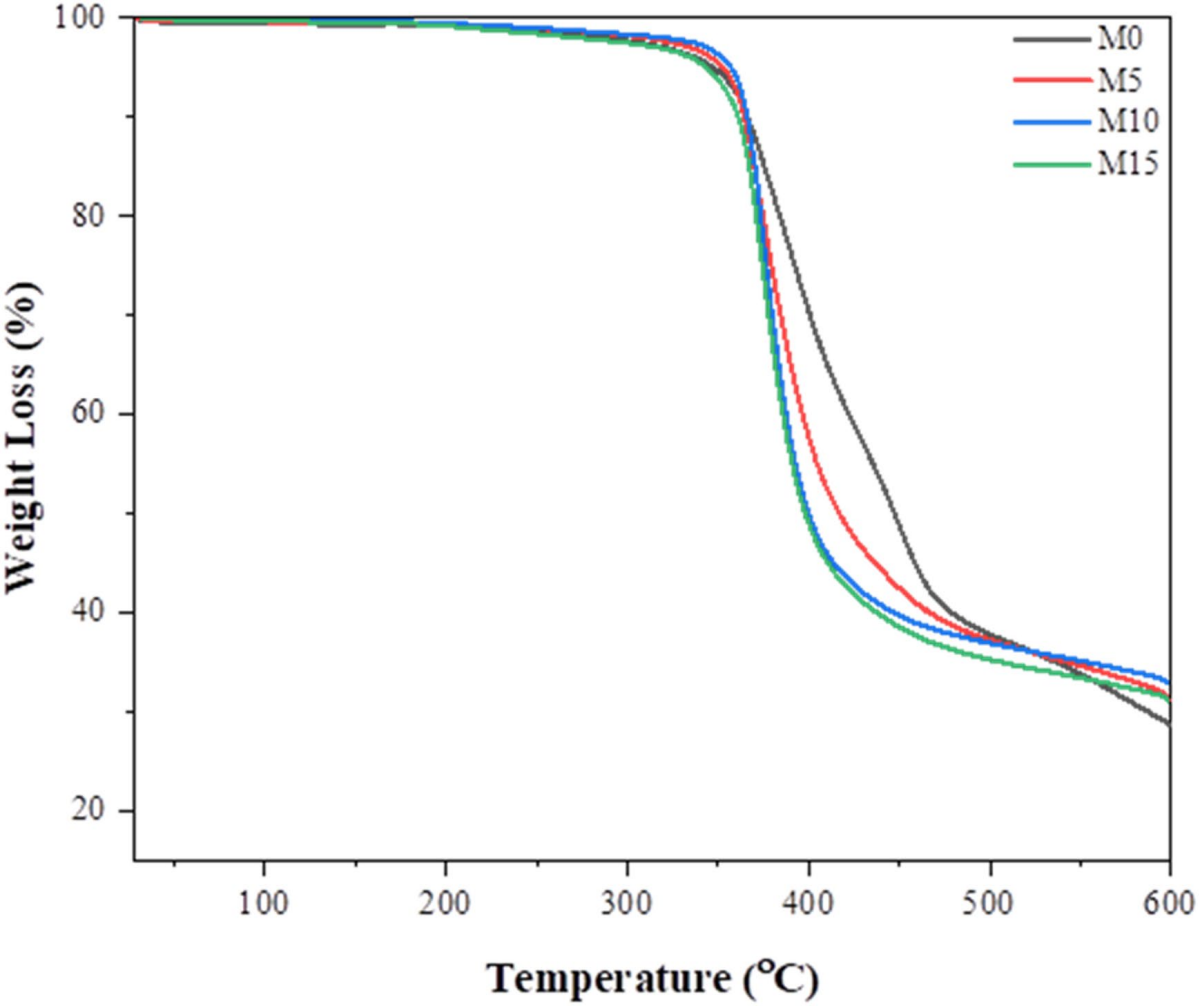
TGA of all membranes along with neat Pebax.

The thermogravimetric curve for all fabricated membranes is shown in Fig. 6. The single thermal weight loss stage seen in the pristine Pebax-1657 membrane in the 350–480 °C range was found due to the thermal decomposition of molecular chains of polymer^21^. The MMMs’ thermal weight loss pattern resembles that of neat Pebax-1657 with a primary thermal breakdown stage located at roughly 360 °C. The MMMs’ primary pyrolysis initiation temperature dropped from 350 to 335 °C as the UiO-66-NH_2_ loading increased. The decrease in intermolecular cohesive energy caused by the additional UiO-66-NH_2_ is thought to be the source of this behavior. The thermal weight loss trend of MMM increased in the range of 380–530 °C. The interference of UiO-66-NH_2_ in the membrane matrix is the reason for these results; yet the MMMs maintained their thermal stability well at high temperatures^18^.

#### SEM

**Fig. 7:**
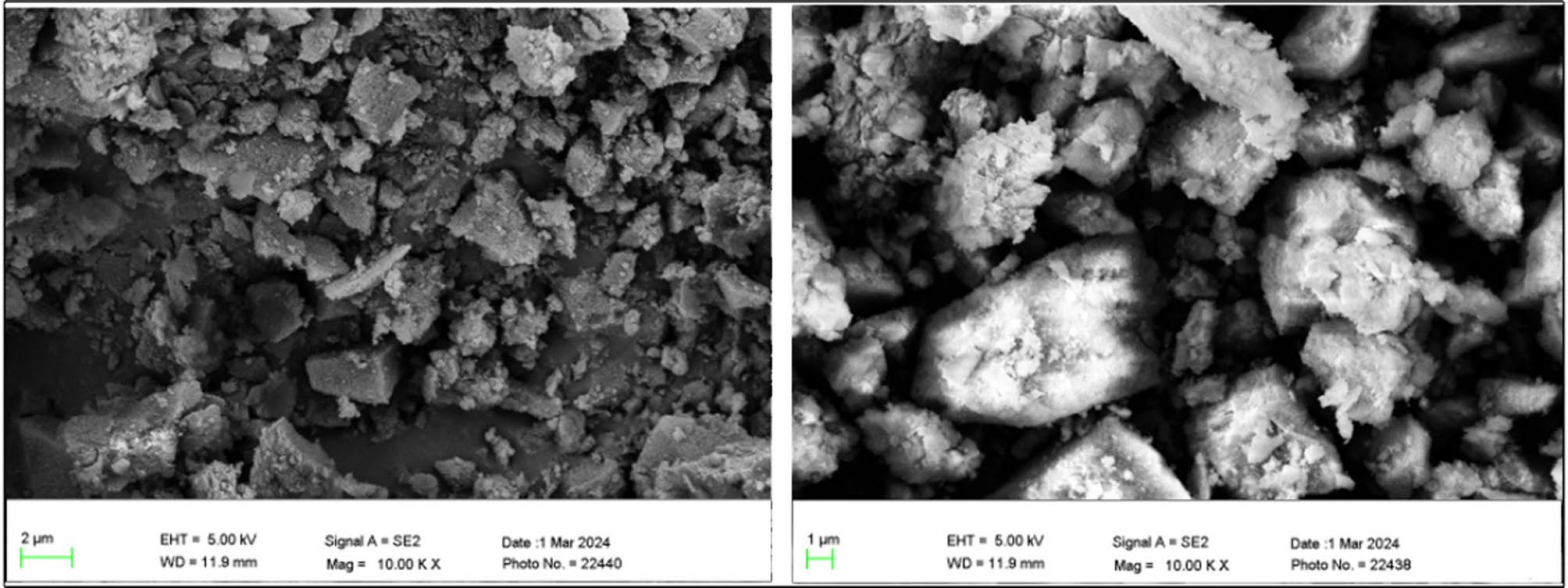
SEM images of UiO-66-NH_2_.

The morphological structures of the prepared UiO-66-NH_2_ are shown in Fig. 7. Unlike previous results, the hydrothermal method yields an octahedral UiO-66-NH_2_ structure. Acetic acid addition can act as an acid modulator that has been found to enhance nucleation and crystal formation, which may be the cause of the lower particle size (~125 nm)^22^.

**Fig. 8:**
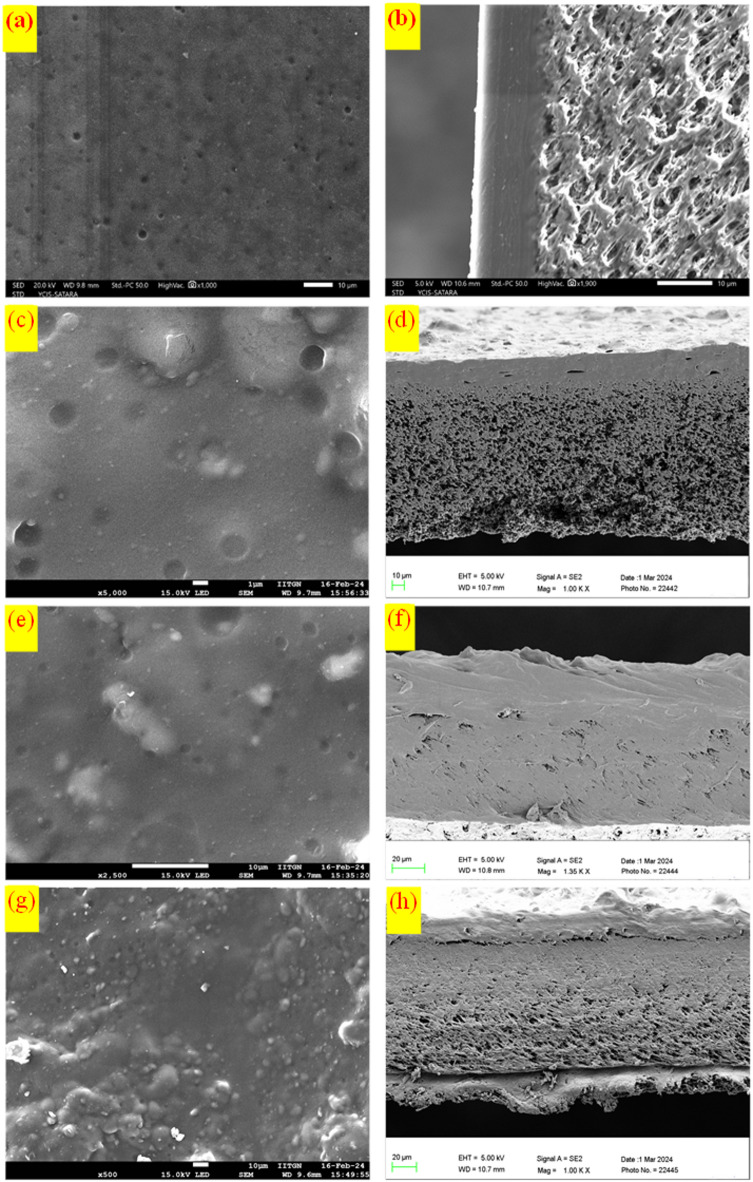
Morphology (left) and cross-sectional (right) images of MMMs; (**a**, **b**) M0, (**c**, **d**) M5, (**e**, **f**) M10, and (**g**, **h**) M15.

The surface and cross-sectional image of pristine Pebax-1657 (M0) and Pebax / UiO-66-NH_2_ / [THTDP][NTf_2_] MMMs is shown in Fig. 8. The morphological images (Fig. 8a, c, e, and g) show that UiO-66-NH_2_ nanoparticles are integrated into the polymer matrix. At 15 wt% loading, the membranes’ surfaces are devoid of noticeable defects. The membrane cross-section pictures clearly demonstrate the nanoparticles’ strong incorporation in the polymer matrix. As its filler loading increases, the surface starts to get a little rough; this could be because of UiO-66-NH_2_. Based on these images, the breadth of every MMM (cast Pebax layer on PVDF) ranges from 10 to 30 μm and is considered for calculating permeability.

### Gas permeation measurements

#### Single gas permeability

**Fig. 9:**
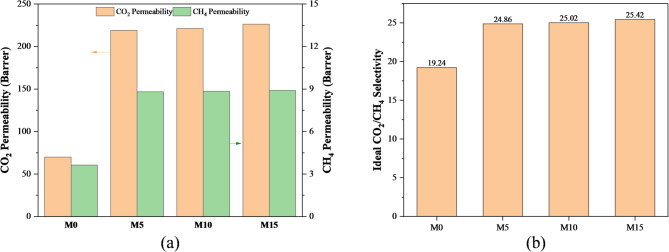
(**a**) Pure gas CO_2_ and CH_4_ permeability, (**b**) Ideal CO_2_/CH_4_ selectivity.

The Single gas selectivity for CO_2_ and CH_4_ and pure CO_2_ and CH_4_ permeabilities for all membranes with different UiO-66-NH_2_ loading at 4 bar and 35 °C are presented in Fig. 9, where the heights of columns indicate the mean value. represents pure gas CO_2_ and CH_4_ permeability and ideal CO_2_/CH_4_ selectivity at 35 °C and 4 bars of feed pressure. It is evident that for all membranes, CO_2_ permeability is far higher than CH_4_ permeability. In general, a combination of kinetic and thermodynamic components is present in gas permeability. Penetrant size and free volume are major determinants of thermodynamic parameters such as gas condensability and its relationship with polymer, as well as kinetic variables^23^. Table 3 presents the characteristics of the gases that are utilized. CO_2_ has greater condensability and gas solubility in membranes than CH_4_ due to its significantly higher critical temperature. The kinetic diameters of molecules of CO_2_ and CH_4_ are 0.33 and 0.38 nm, in that order. As a result, CO_2_ has a greater diffusion coefficient than CH_4_. Because of its high condensability and lower kinetic diameter, CO_2_ has a larger permeability. For better understanding of the adsorption property of gas on UiO-66-NH2 particle N_2_ gas adsorption experiment (BET analysis) has been done, and detailed description has been provided in supplementary file (Figs. S1–S3). N_2_ adsorption–desorption analysis at 77 K revealed a typical Type I isotherm, confirming the microporous nature of UiO-66-NH_2_. The nitrogen adsorption–desorption isotherm exhibited a typical Type I profile, confirming the microporous nature of the material. The high BET surface area (555.8 m^2^ g^−1^) and significant micropore volume (0.137 cc g^−1^) indicate abundant accessible adsorption sites. The average pore diameter (~ 2.3 nm) lies within the microporous range, which is favorable for enhanced gas–solid interactions and confinement effects. Furthermore, the presence of amine functional groups in UiO-66-NH_2_ is expected to promote strong interactions with CO_2_ molecules through acid–base and quadrupole interactions. Therefore, the textural and chemical properties suggest that the material is promising for CO_2_ adsorption applications.

The loading range was intentionally selected for three reasons. First, several studies on UiO-type MOF/Pebax MMMs have shown that relatively low MOF contents (typically ≤ 10 wt%) are often sufficient to capture most of the beneficial effect on CO_2_ transport. Second, higher MOF contents tend to promote particle agglomeration and interfacial defects (non-selective voids), which can deteriorate selectivity and mechanical integrity; this behaviour has been explicitly reported for UiO-66-based MMMs and other MOF/Pebax systems where increasing loading beyond an optimal value reduced CO_2_/CH_4_ or CO_2_/N_2_ selectivity due to aggregation and poor polymer–filler contact. Third, for our specific Pebax-1657/UiO-66-NH_2_/[THTDP][NTf_2_] system, maintaining good film-forming ability and avoiding brittleness was critical, and preliminary trials with higher UiO-66-NH_2_ contents showed processing difficulties and defect formation (cracks, local agglomerates), so we limited the study to 15 wt% as a practical upper bound. For these reasons, the main purpose of exploring 5, 10, and 15 wt% UiO-66-NH_2_ was not to seek a monotonic improvement with loading, but to identify a practically achievable window where (i) the MOF is well dispersed, (ii) the membrane remains defect-free and mechanically robust, and (iii) the CO_2_/CH_4_ performance moves closer to the Robeson trade-off without sacrificing processability^24–27^. The permeability of CO_2_ and CH_4_ rises in all MMMs with increasing UiO-66-NH_2_ loading. For example, the CO_2_ and CH_4_ permeabilities of M15, which has a loading of 15 wt% of UiO-66-NH_2_, are 226.37 and 8.9 Barrer, respectively. The CO_2_ permeability is observed to be approximately 223% enhanced when compared to the pure Pebax-1657 membrane. The increased free volume resulting from the disturbed initial polymer chain packing around the interface may be the reason for the enhancement in permeability values seen with increasing UiO-66-NH_2_ loading. Because CO_2_ molecules are more concentrated in the membrane matrix than light gases are, free volume increase has a major role in increasing CO_2_ permeability^28^. Because CO_2_ and NH_2_ are Lewis acid-base compounds, the -NH_2_ offers more active sites for CO_2_ adsorption, allowing for strong electrostatic interactions. The improvement of CO_2_ affinity for UiO-66-NH_2_ is the outcome of this interaction. As a result, this enhances CO_2_ and CH_4_ separation efficiency overall^29^. In addition, [THTDP][NTf_2_], which has a phosphonium-based cation, possesses significantly higher CO_2_ solubility than most imidazolium-based ionic liquids. Notable benefits of the [NTf_2_] anion include its good selectivity and CO_2_ solubility^30^, as well as its good thermal stability and compatibility with polymers^31^. When compared to CH_4_, these characteristics significantly increase CO_2_ permeability. Higher ideal CO_2_/CH_4_ selectivity results from this improvement (Fig. 9b). The CO_2_/CH_4_ selectivity for M15 is 25.42, around 32% greater compared to the pristine Pebax-1657 membrane. The notable efficacy of the M15 is indicated by the simultaneous improvement of gas permeability and ideal gas selectivity in this work, as previously discussed.

**Table 2:** Permeability and selectivity of Pure CO_2_ and CH_4_ gases in fabricated membranes.

Membrane	Permeability (CO_2_) (a)	Permeability (CH_4_) (b)	Selectivity (a/b)
M0	70.02	3.64	19.24
M5	219.1	8.81	24.86
M10	221.24	8.84	25.02
M15	226.37	8.9	25.42

**Table 3:** Physical properties of carbon dioxide and methane^28^.

Gas	Kinetic diameter (nm)	Quadrupole moment (g^0.5^.cm^3.5^.s^− 1^ × 10^26^)	Critical temperature T_c_ (°C)
CO_2_	0.33	−4.3	31.1
CH_4_	0.38	0	−82.6

#### Mixed gas permeation

The competitive absorption of penetrants, gas phase nonideality, and potential plasticization caused by condensable gases like CO_2_ are the main causes of the differences in permeation results between pure and mixed gas studies^32^. In this context, MMMs with different UiO-66-NH_2_ loadings have been studied at different CO_2_ partial pressures with a CO_2_:CH_4_ ratio of 30:70% v/v.

**Fig. 10:**
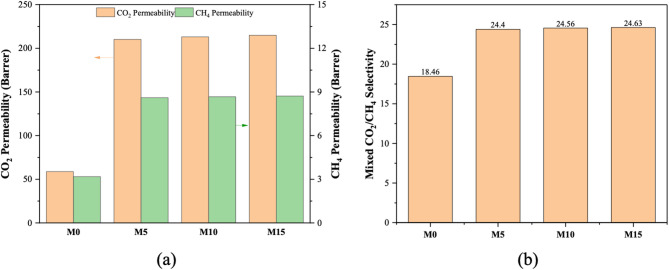
(**a**) Mixed gas CO_2_ and CH_4_ permeability, (**b**) Mixed CO_2_/CH_4_ selectivity.

The purpose of this study is to examine how UiO-66-NH_2_ loading affects CO_2_ passage through it and its separation from mixed gas. For all membranes with varying UiO-66-NH_2_ loading at 4 bars of total input pressure and 35 °C, Fig. 10 shows mean values of mixed CO_2_/CH_4_ selectivity and mixed CO_2_ and CH_4_ permeabilities. The mixed gas CO_2_/CH_4_ permeability and mixed CO_2_/CH_4_ selectivity at 35 °C and 4 bars of total pressure at the feed are shown in Table 4. The permeability of CO_2_ and CH_4_ rises in all MMMs with increasing UiO-66-NH_2_ loading. For example, the CO_2_ and CH_4_ permeabilities of M15, which has a loading of 15 wt% of UiO-66-NH_2_, are 215.04 and 8.72 Barrer, respectively. The CO_2_ permeability is observed to be approximately 265% enhanced when compared to the pure Pebax-1657 membrane. The CO_2_/CH_4_ selectivity for M15 is 24.63, around 33% greater than that of a pristine Pebax-1657 membrane. It is worth noting that CO_2_ permeability and separation factors are both lower for the mixed gas study compared to the pure gas investigation. The reason behind this behavior is the presence of CH_4_ in the gas. As CH_4_ gradually increases in gas flux, this leads to a reduction in CO_2_ permeability.

**Table 4:** Permeability and selectivity of mixed CO_2_ and CH_4_ gases in fabricated membranes.

Membrane	Permeability (CO_2_) (a)	Permeability (CH_4_) (b)	Selectivity (a/b)
M0	58.79	3.18	18.46
M5	210.18	8.61	24.40
M10	213.08	8.67	24.56
M15	215.04	8.72	24.63

#### Effect of feed pressure

The properties and performances of membranes for pressure-dependent gas separation must be understood to design an efficient membrane for gas separation applications. It is crucial to examine the impact of pressure on MMMs since the adsorption properties of additives and the polymer cross-link configuration significantly affect gas transport^33^. Here, the MMMs’ separation performance was investigated at a driving pressure of 1 bar to 4 bar.

**Fig. 11:**
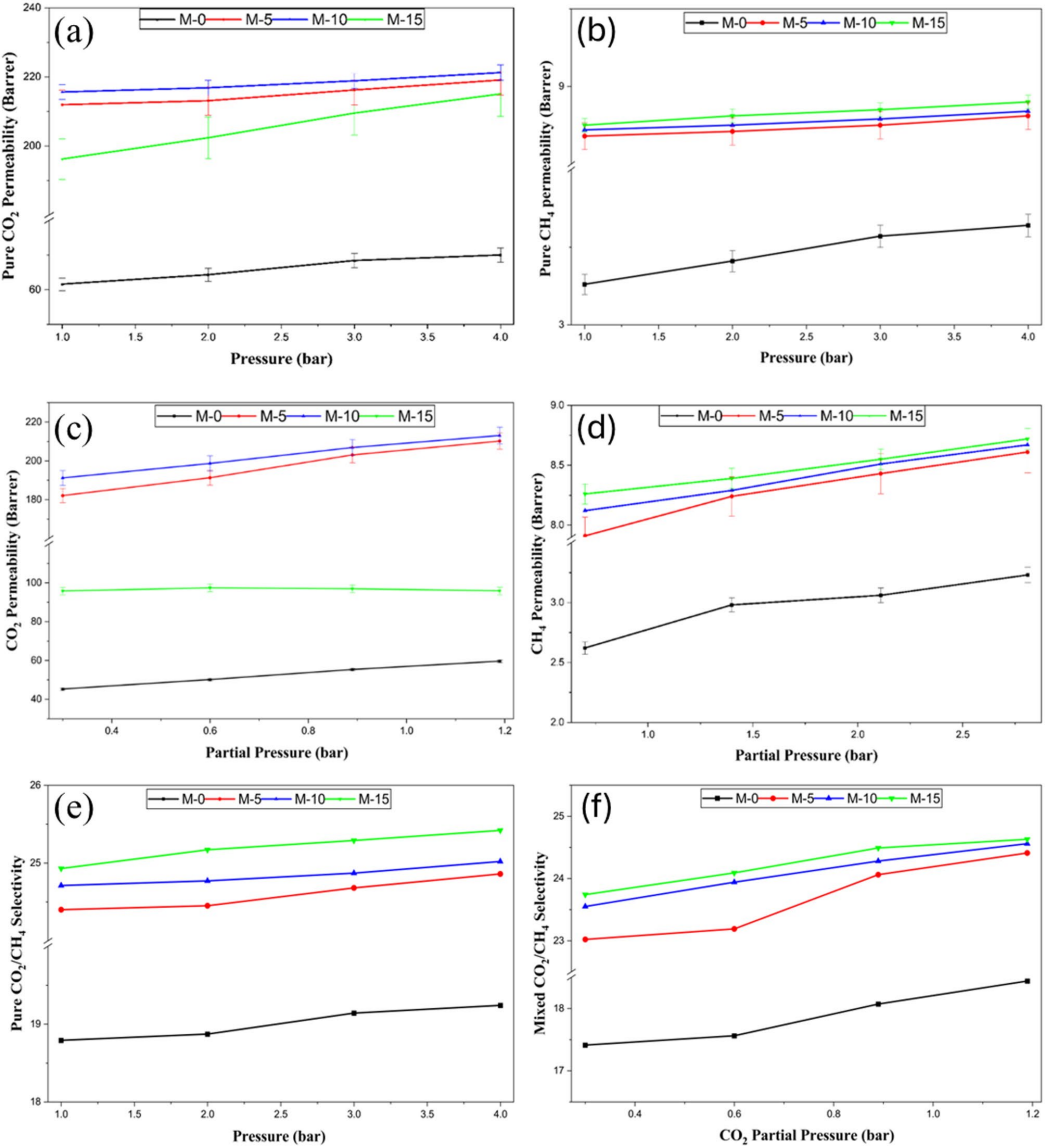
Permeability and selectivity of MMMs. (**a**), (**b**), (**c**), and (**d**) indicated pure and mixed gas permeabilities, (**e**) and (**f**) ideal and mixed selectivity’s.

The effect of feed pressure on CO_2_/CH_4_ selectivity and CO_2_ and CH_4_ permeability of all MMMs and pristine Pebax-1657 membranes for mixed and pure gas studies is shown in Fig. 11. For the mixed gas study, the ratio of CH_4_ to CO_2_ is taken to be 70:30%v/v. It is evident from the graphs in Fig. 11a, b, c, and d that permeability rises as pressure does. This can be explained by the fact that molecules on the permeate side may begin to flow through the crystal pores as a result of the packing effect of pressure, reducing some of the interface gaps. The rise in CO_2_ permeability suggests that the hydrostatic pressure effect is overcome by an increased number of condensable CO_2_ molecules, increasing the free volume of the membrane. Moreover, CO_2_ can raise the polymer chain’s mobility in the case of a pristine membrane, which causes the polymer to plasticize^34^. In contrast, UiO-66-NH_2_’s adsorption capabilities boost the gas permeability for MMMs. According to^35^, the adsorbed amounts of CO_2_ increase significantly as pressure rises (that is, the adsorbed quantity of CO_2_ increases ninefold when pressure rises from 1 bar to 10 bar). The impact of feed pressure on the ideal and mixed CO_2_/CH_4_ selectivity for each membrane is shown in Fig. 11e and f. As can be seen, in both situations, CO_2_/CH_4_ selectivity rises as pressure rises. Permeability and selectivity in mixed gas are both poorer than in pure gas research due to competitive sorption. Supplementary file Tables S1 and S2 indicates the entire values of permeability and selectivity with pressure for both pure and mixed gases.

#### Performance of MMMs

The impact of ILs on MOF-based MMM was investigated by Vu et al.^36^. They used the following ILs for this purpose: 1-ethyl-3-methylimidazolium bis (trifluoromethanesulfonyl) imide ([Emim][NTf_2_]), 1-butyl-3-methylimidazolium tetrafluoroborate ([Bmim][Bf_4_]), and 1-butyl-3-methylimidazolium bis (trifluoromethanesulfonyl) imide ([Bmim][NTf_2_]), all of which were combined with ZIF-67 as MOF and 6FDA-durene as polymer. MMM with 20 wt% [Emim][NTf_2_]@HKUST-1/PI demonstrated the most effective gas separation performance. The CO_2_/CH_4_ selectivity and CO_2_ permeability both significantly improved, increasing by about 28% and 112%, respectively. This improvement may be attributable to a decrease in interfacial voids between ZIF-67/IL/PI MMMs compared to ZIF-67/PI MMMs. [Bmim][NTf_2_] was utilised by Li et al.^37^ to modify ZIF-8 and fabricate [Bmim][NTf_2_]@ZIF-8/Pebax MMMs. The enhanced suitability between ZIF-8 and Pebax led to an improvement in permeability and selectivity of CO_2_ from a CO_2_/CH_4_ gas mixture to 45% and 92%, respectively, as compared to the neat Pebax membrane. Li et al.^38^ created MMMs using [Bmim][NTf_2_]@ZIF-8/Pebax with a 15 wt% filler loading in their work, and through gas tests, they discovered improvements in CO_2_ permeability and selectivity to 1.6% and 4.6%, respectively. This improvement can be due to ZIF-8 and [Bmim][NTf_2_] working in harmony. [Emim][NTf_2_]@HKUST-1/PI MMMs were examined the CO_2_/CH_4_ and CO_2_/N_2_ separation capabilities in the study conducted by Lin et al.^39^. [Emim][NTf_2_] can decrease interfacial voids and enhance CO_2_ selectivity, according to the results. MMM with 10 wt% [Emim][NTf_2_]@HKUST-1/PI improved CO_2_/CH_4_ selectivity by about 22% and CO_2_ permeability by around 41%, respectively. Table 5 summarizes these comparative data.

**Table 5:** Separation performance of CO_2_ from CO_2_/CH_4_ mixture various mixed matrix membranes with UiO-66-NH_2_ as filler.

Polymer	IL	Filler	Loading (wt%)	Parameter [*P*(Bar) & T(ºC)	*P*_CO2_ (Barrer)	CO_2_/CH_4_ Selectivity	References
Pebax-1657	[THTDP][NTf_2_]	–	–	4, 35	70.02	19.24	This work
		UiO-66-NH_2_	15	4, 35	226.37	25.42	
Pebax-1657	[Bmim][NTf_2_]	–	–	1, 25	70	18.1	^37^
		ZIF-8	15	1, 25	104.9	34.8	
Pebax-1657	[Bmim][NTf_2_]	–	–	1, 23	110	16.8	^38^
		ZIF-8	15	1, 23	111.8	17.5	
6FDA-durene	[Emim][NTf_2_]	–	–	2, 25	780	24.1	^39^
		HKUST-1	10	2, 25	1102	29.3	
6FDA-durene		–	––	2 atm, 25	670	19.9	^36^
	[Bmim][Bf_4_]	ZIF-67	20		1350	24	
	[Emim][NTf_2_]				1420	25.5	
	[Bmim][NTf_2_]				895	28	

**Fig. 12:**
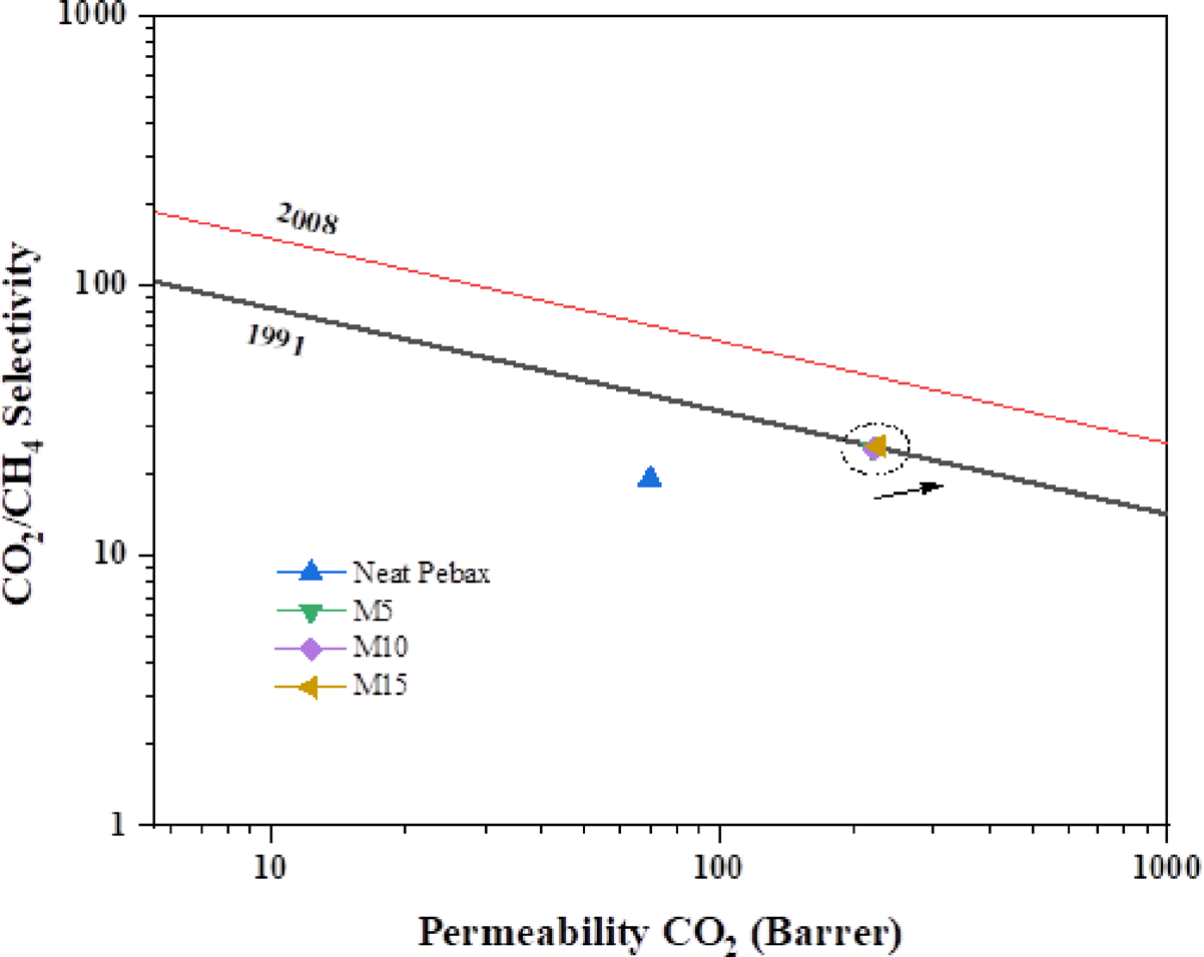
CO_2_/CH_4_ Robeson upper bond of Pebax/UiO-66-NH_2_/[THTDP][NTf_2_] MMMs.

All the fabricated MMMs’ CO_2_/CH_4_ gas separation performance is displayed in Fig. 12. Among the others, the MMM with 15 wt% of UiO-66-NH_2_ performs the best in CO_2_/CH_4_ separation. Every Pebax-1657/UiO-66-NH_2_/[THTDP][NTf_2_] MMM crosses the 1991 Robeson upper bond^40,41^. It has also been shown that CO_2_ permeability and CO_2_/CH_4_ selectivity both rise with filler loading, which ranges from 5 to 15 wt%. This pattern outperforms the trade-off relationship between the two variables. The addition of the UiO-66-NH_2_ nanoparticles and [THTDP][NTf_2_] surpasses the 1991 upper bound and reaches close to the 2008 upper bound, indicating the performance of the prepared MMMs.

## Conclusions

The current work examined the effect of UiO-66-NH_2_ microporous MOFs and [THTDP][NTf_2_] on the gas transport characteristics of MMMs based on Pebax-1657. Hydrothermal treatment was utilized to synthesize UiO-66-NH_2_ nanoparticles. The MOFs loadings for the MMMs ranged from 5 to 15 wt%. SEM images showed that the nanoparticles were well dispersed throughout the polymer matrix. The polymer’s gas permeability was enhanced by the breaking of interchain bonds within the Pebax-1657 matrix, as seen by the XRD spectra. The thermal endurance of membranes improves when UiO-66-NH_2_ loading increases, according to TGA data. Experiments on single and mixed gas permeation were carried out to assess the performance of the MMMs. The findings demonstrate that, as UiO-66-NH_2_ loading increases, CO_2_ and CH_4_ permeability rise because of the nanoparticles’ extra free volume and the polymer chain expansion. Furthermore, there is a noticeable improvement in the ideal selectivity as the UiO-66-NH_2_ loading increases. At a total of four different pressures (1, 2, 3, and 4 bar), the effect of transmembrane pressure on the separation of CO_2_ from CO_2_/CH_4_ using the membrane is studied. As the pressure increased, all membranes exhibited increased CO_2_ and CH_4_ permeability. As a result, membrane performance increased with feed pressure. The MMMs showed a notable improvement in CO_2_ separation efficiency by crossing the Robeson upper bound.

## Supplementary Information

Below is the link to the electronic supplementary material.

Supplementary Material 1

## Data Availability

The datasets used and analysed during the current study are available from the main corresponding author (Dr. Swapnil Dharaskar) on reasonable request.
