# Supplementary material for: Synergetic effect of phosphonium-based ionic liquid and amine-functionalized metal organic framework for separation of CO2 and CH4 using mixed matrix membranes
Source: Sci Rep. 2026 Apr 13;16:17308. doi: 10.1038/s41598-026-48922-4 (PMC13234182; doi:10.1038/s41598-026-48922-4)
Supplement: Supplementary file 1 — Supplementary Material 1 [file 41598_2026_48922_MOESM1_ESM.pdf]

Supplementary File

Journal: **Scientific Reports**
Manuscript Number: **eb8f4a8b-3d44-4dea-9f4d-9a1208f9cf32**

Title: "***Synergetic effect of Phosphonium-based Ionic Liquid and Amine-Functionalized Metal Organic Framework for Separation of CO_2 and CH_4 using Mixed Matrix Membranes***".

BET of Uio-66-NH_2

Nitrogen adsorption–desorption analysis was performed at 77 K to evaluate the textural properties of UiO-66-NH_2. The adsorption–desorption isotherm (pages 2–3) shows a steep nitrogen uptake at low relative pressure ($P/P_0 < 0.1$), indicating the presence of dominant microporosity. The isotherm exhibits characteristics of a Type I profile, which is typical for microporous metal–organic frameworks. The multipoint BET analysis (page 4) gave a specific surface area of 555.78 $m^2\ g^{-1}$ with a high correlation coefficient ($r = 1$), confirming good linear fitting in the selected pressure range. The BET constant ($C = 592.93$) is relatively high, suggesting strong interaction between nitrogen molecules and the framework surface. The Langmuir surface area (page 6) was calculated as 738.65 $m^2\ g^{-1}$, which is typically higher than the BET value due to the monolayer adsorption assumption. The t-plot analysis (page 7) revealed a micropore volume of 0.1372 cc g^{-1} and a micropore surface area of 329.33 $m^2\ g^{-1}$, confirming that micropores significantly contribute to the total surface area. The external surface area was calculated as 226.45 $m^2\ g^{-1}$.Pore size distribution obtained from the DFT method (pages 10–12) showed a dominant pore width centred around ~2.03 nm, with a total pore volume of 0.3052 cc g^{-1} and a DFT surface area of 621.82 $m^2\ g^{-1}$. The average pore diameter was found to be approximately 2.31 nm, which is consistent with the expected microporous structure of UiO-66-NH_2.

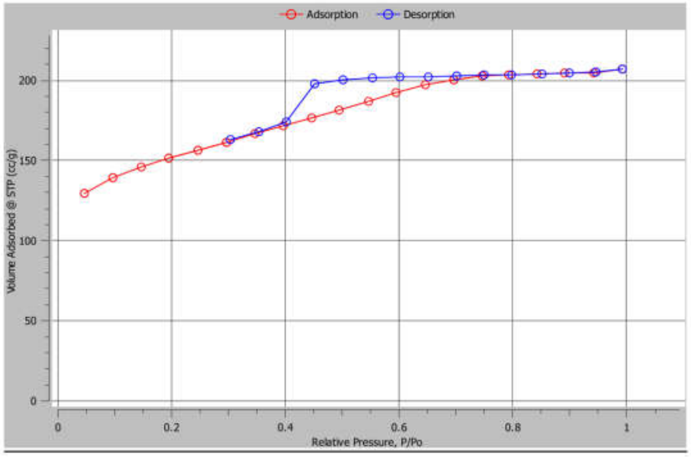

Figure S 1 : N2 adsorption desorption diagram of UiO-66-NH_2

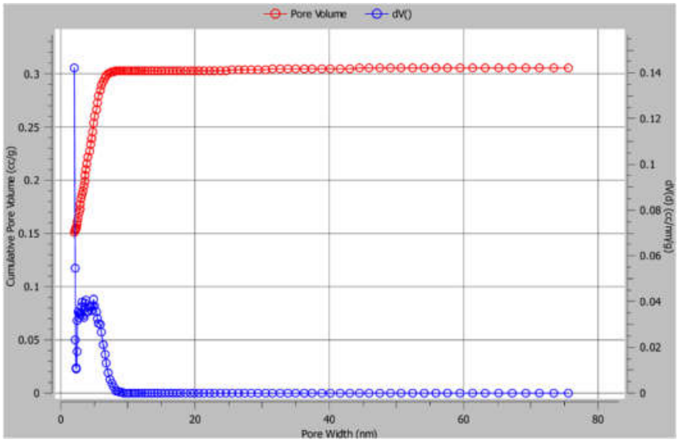

Figure S 2 :Pore size distribution DFT method

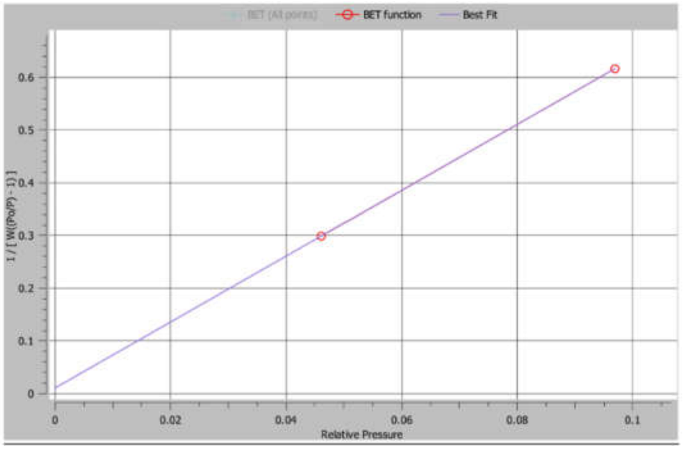

**Isotherm Branch**	Adsorption	**Slope**	6.25544	**Intercept**	0.0105678
**Correlation coeff., r**	1	**C constant**	592.932	**Surface area**	555.779 m²/g

Figure S 3 : Multiple point BET of UiO-66-NH2

Table S1 Pure gas permeability and selectivity of CO_2 and CH_4

Pressure	Pure CO_2 Permeability (Barrer)				Pure CH_4 Permeability (Barrer)				Selectivity (Pure gas)			
(bar)	M0	M-5	M-10	M-15	M-0	M-5	M-10	M-15	M-0	M-5	M-10	M-15
1	61.58	211.94	215.61	218.18	3.26	8.68	8.72	8.75	18.79	24.4	24.71	24.93
2	64.32	213.1	216.84	222.01	3.41	8.71	8.75	8.81	18.87	24.45	24.77	25.17
3	68.42	216.2	218.85	224.06	3.57	8.75	8.79	8.85	19.14	24.68	24.87	25.29
4	70.02	219.1	221.24	226.37	3.64	8.81	8.84	8.9	19.24	24.86	25.02	25.42

Table S2 Mixed gas permeability and selectivity of CO_2 and CH_4

Press	Mixed CO_2 Permeability (Barrer)				Press	Mixed CH_4 Permeability (Barrer)				Press	Selectivity			
(bar)	M-0	M-5	M-10	M-15	(bar)	M-0	M-5	M-10	M-15	(bar)	M-0	M-5	M-10	M-15
0.3	45.21	182.07	191.22	95.73	0.7	2.62	57.91	8.12	8.26	0.3	17.41	23.02	23.55	23.74
0.6	50.07	191.31	198.65	97.37	1.4	2.98	8.24	8.29	8.39	0.6	17.56	23.19	23.94	24.09
0.89	55.28	203.06	206.86	96.88	2.11	3.06	8.43	8.51	8.55	0.89	18.07	24.06	24.28	24.49
1.19	59.58	210.19	213.08	95.82	2.81	3.23	8.61	8.67	8.72	1.19	18.44	24.41	24.56	24.63

Table S3 Standard Deviation Values of Mixed Gas Permeability

	Pressure(bar)	M0	M-5	M-10	M-15
Mixed CO_2	0.3	0.0371	0.0413	0.0107	0.0218
	0.6	0.0100	0.0115	0.0074	0.0183
	0.89	0.0055	0.0101	0.0082	0.0044
	1.19	0.0102	0.0193	0.0082	0.0198
Mixed CH_4	0.7	0.0037	0.0092	0.0874	0.0101
	1.4	0.0009	0.0045	0.0161	0.0089
	2.11	0.0036	0.0067	0.0044	0.0037
	2.81	0.0054	0.0027	0.0019	0.0042

Table S4 Standard Deviation Values of Pure Gas Permeability

	Pressure(bar)	M0	M-5	M-10	M-15
Pure CO_2	1	0.0035	0.4553	0.0212	0.0098
	2	0.0040	0.2938	0.0063	0.0056
	3	0.0021	0.4879	0.0028	0.0059
	4	0.0012	0.2919	0.0038	0.0061
Pure CH_4	1	0.0032	0.3619	0.0116	0.0060
	2	0.0035	0.0466	0.0037	0.0087
	3	0.0040	0.0234	0.0045	0.0033
	4	0.0022	0.0028	0.0036	0.0021

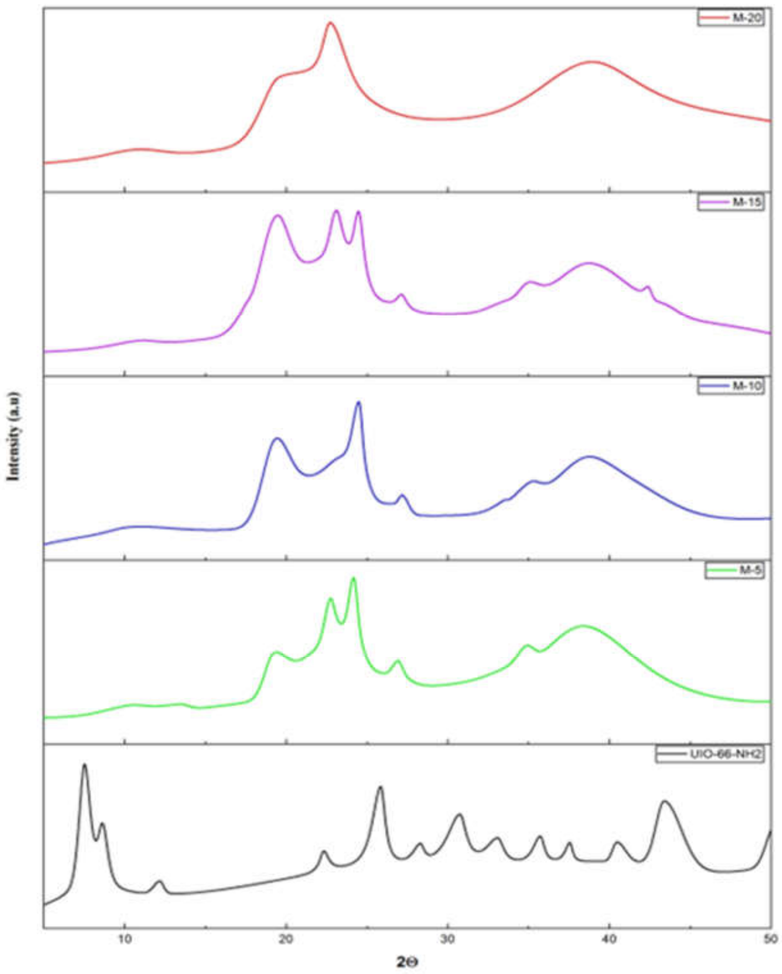

Figure S 4 XRD Plot of Uio-66-NH_2 Particle along with Membranes
